# Supplementary material for: Sarcopenia Is Associated with Changes in Circulating Markers of Antioxidant/Oxidant Balance and Innate Immune Response
Source: Antioxidants (Basel). 2023 Nov 11;12(11):1992. doi: 10.3390/antiox12111992 (PMC10669556; doi:10.3390/antiox12111992)
Supplement: Supplementary file 1 [file antioxidants-12-01992-s001.zip › antioxidants-2661609-supplementary.pdf]

Supplementary Material

Article

Sarcopenia is associated with changes in circulating markers of antioxidant/oxidant balance and innate immune response

Francesco Bellanti, Aurelio Lo Buglio, Stefano Quiete, and Gianluigi Vendemiale

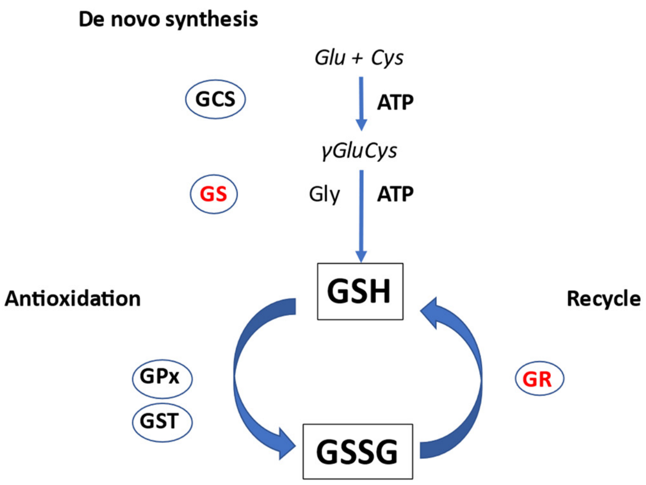

Figure S1. Schematic representation of the glutathione pathway. Glutathione synthesis is a two-step process catalyzed by the enzyme γ-glutamylcysteine synthetase (GCS) and glutathione synthetase (GS). Reduced glutathione (GSH) scavenges reactive species via glutathione peroxidase (GPx) and glutathione S-transferase (GST) and becomes oxidized (GSSH). Recycling of GSSG to GSH is catalyzed by glutathione reductase (GR).

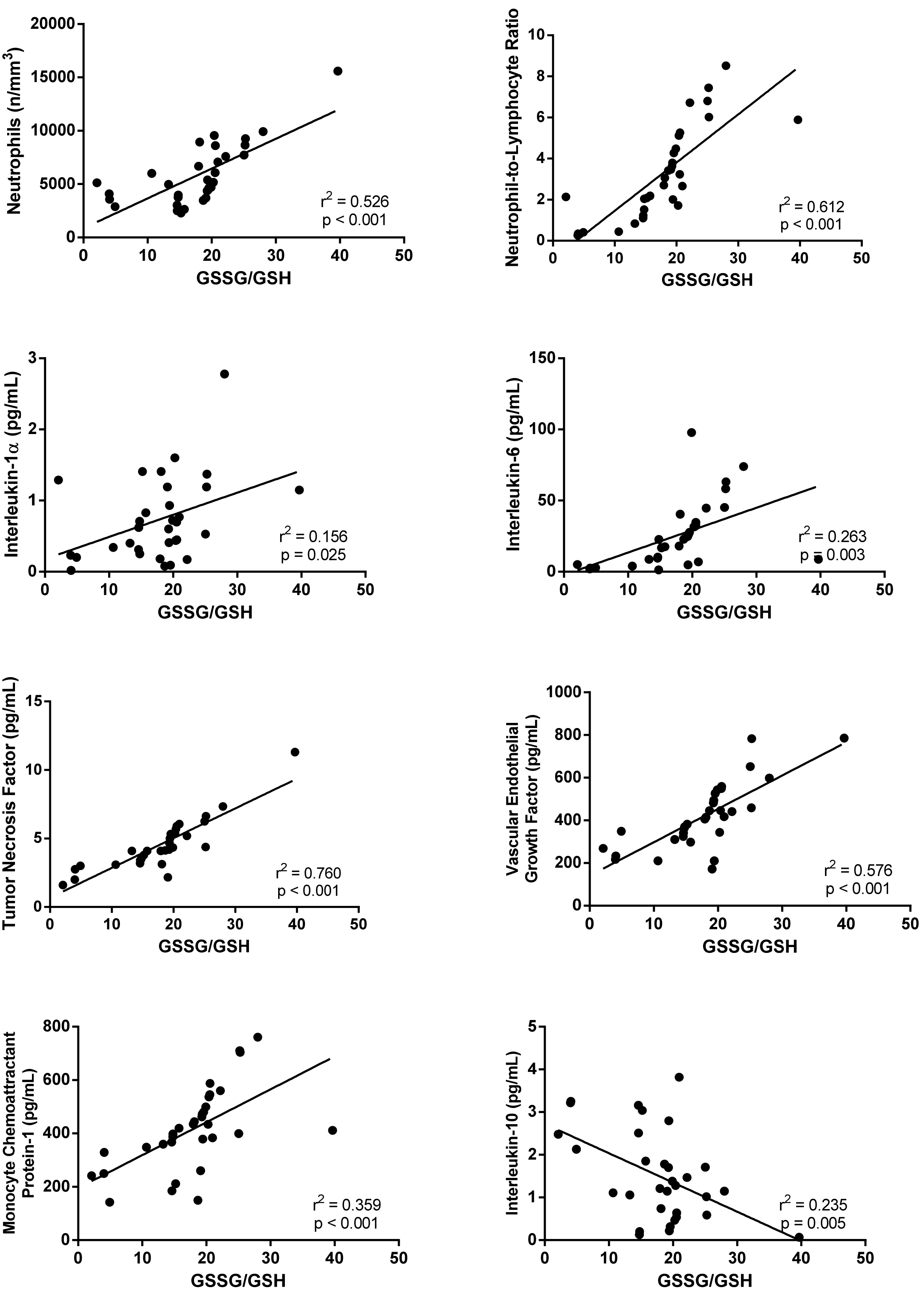

Figure S2. Linear regression analysis between blood oxidized (GSSG)/reduced (GSH) glutathione ratio and circulating markers of immune response in sarcopenic patients.

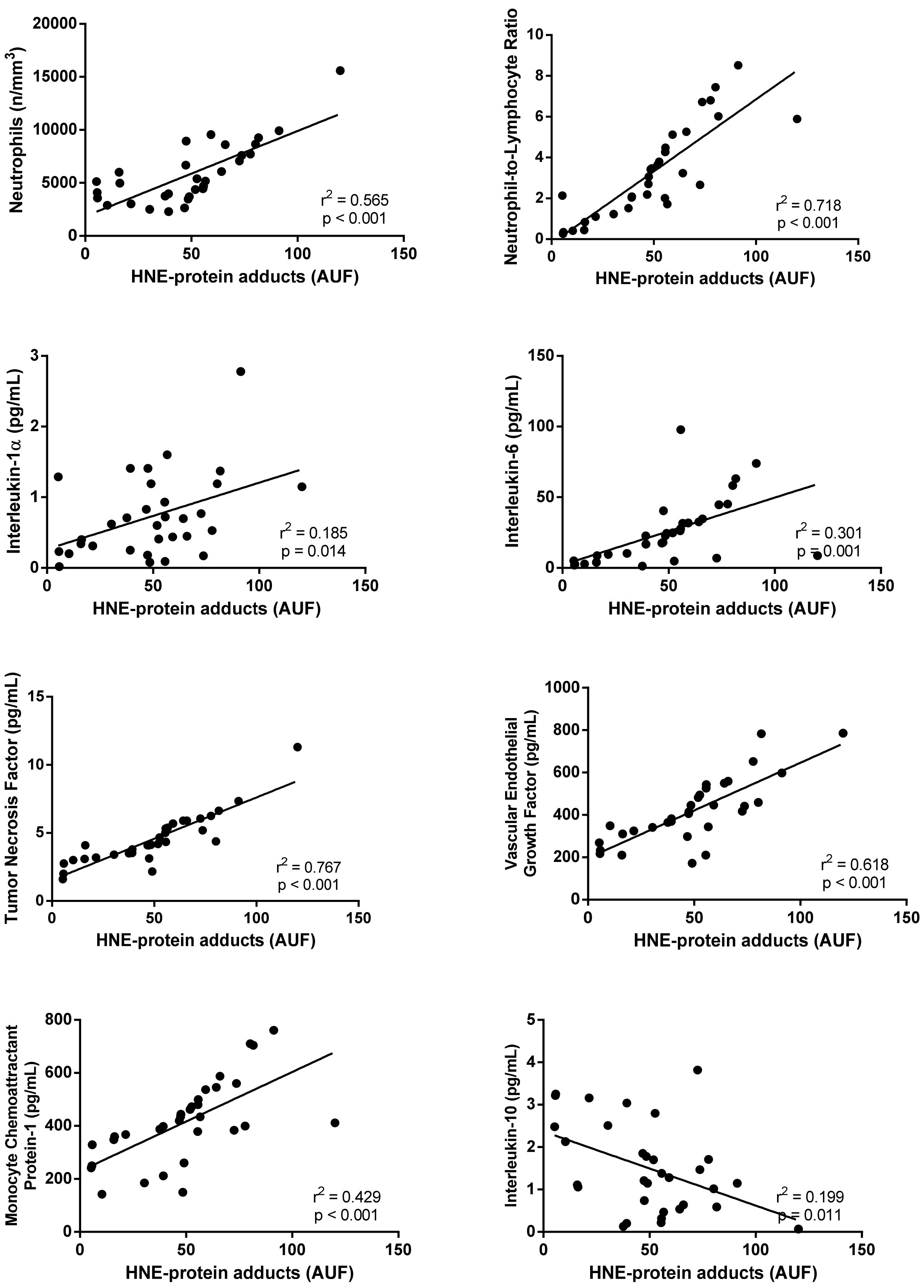

Figure S3. Linear regression analysis between serum hydroxynonenal (HNE)-protein adducts and circulating markers of immune response in sarcopenic patients. AUF, arbitrary units of fluorescence.

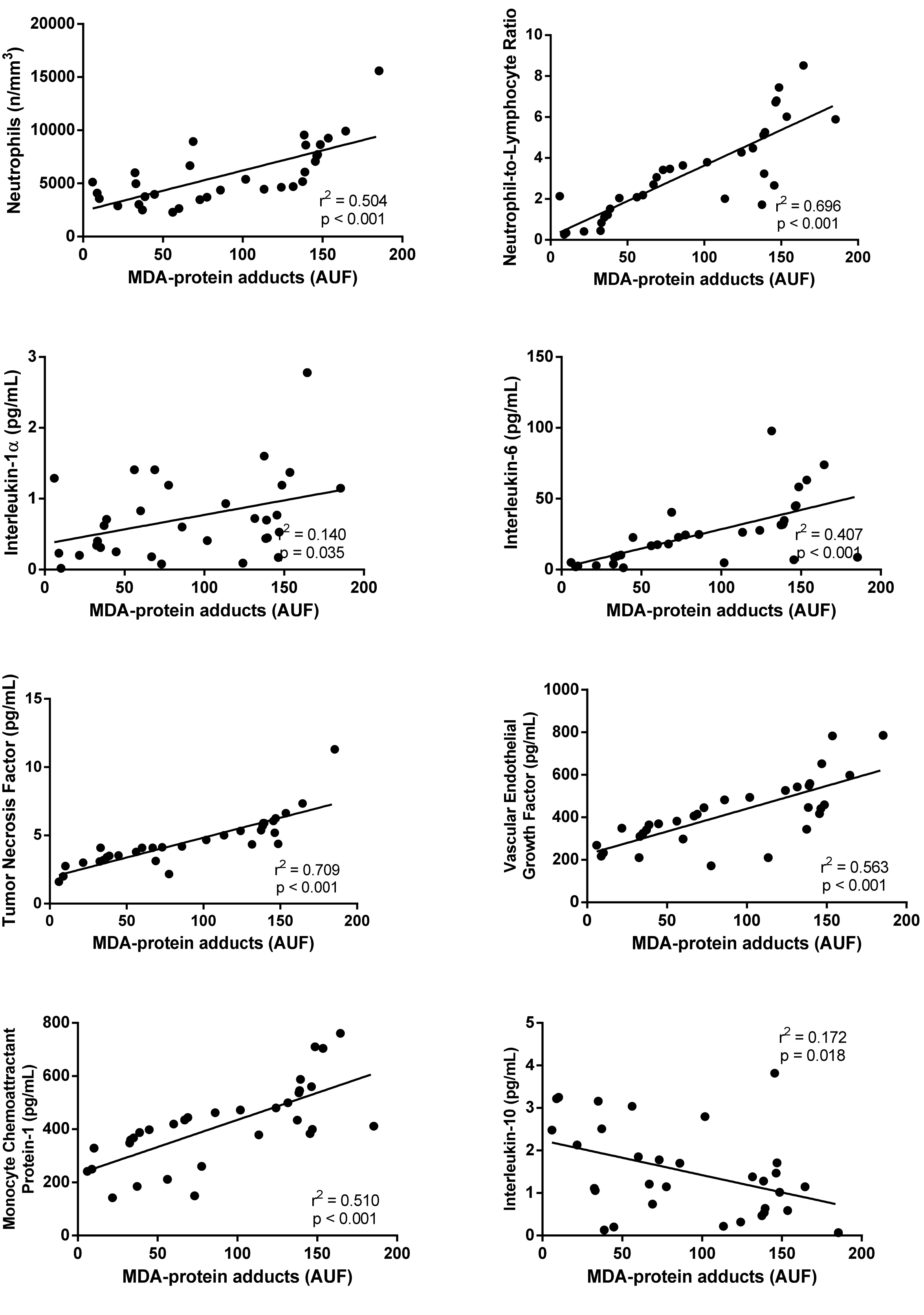

Figure S4. Linear regression analysis between serum malondialdehyde (MDA)-protein adducts and circulating markers of immune response in sarcopenic patients. AUF, arbitrary units of fluorescence.
